# Gastric Duplication Cyst Revealed After an Endoscopic Ultrasound-Guided Fine-Needle Aspiration of a Suspected Mucinous Cystadenoma of the Pancreas

**DOI:** 10.7759/cureus.19560

**Published:** 2021-11-14

**Authors:** Yasmine Cherouaqi, Fatima Belabbes, Mohammed Allaoui, Abderahmane Al Bouzidi, Fedoua Rouibaa

**Affiliations:** 1 Gastroenterology and Proctology, Faculty of Medicine, Mohammed VI University of Health Sciences (UM6SS) Cheich Khalifa International University Hospital, Casablanca, MAR; 2 Department of Pathology, Military General Hospital, Rabat, MAR; 3 Pathology, Faculty of Medicine, Mohammed VI University of Health Sciences (UM6SS) Cheich Khalifa International University Hospital, Casablanca, MAR

**Keywords:** diagnosis, gastrointestinal duplication, endoscopic ultrasound, duplication cyst, gastric duplication cyst

## Abstract

Gastrointestinal duplication is a rare congenital anomaly of the gastrointestinal tract. Gastric duplication cysts (GDCs) are uncommon in adults, and most cases are discovered incidentally. Here, we report a fortuitous discovery of a rare case of an asymptomatic noncommunicating GDC in an adult revealed after an endoscopic ultrasound-guided fine-needle aspiration of a suspected mucinous cystadenoma of the pancreas. A 34-year-old female presented with renal colic. Her abdominal examination was normal. She presented a cystic image at the left lumbar discovered fortuitously during ultrasonography. On uro-computed tomography, there was a suspicion of a pancreatic cystadenoma. Magnetic resonance imaging of the pancreas suggested a mucinous cystadenoma of the pancreatic tail. The endoscopic ultrasound showed a cystic thick-walled formation in the tail of the pancreas. After guided fine-needle aspiration, a split aspect of the gastric wall appeared evoking a GDC. The cytology showed epithelial cells without mucin. Three years later, the patient does not have any gastrointestinal symptoms. GDCs are a rare anomaly, and accurate diagnosis of these cysts is difficult. Surgical resection can offer a definitive diagnosis. The mainstay of treatment is surgery to avoid the risk of malignancy.

## Introduction

Gastrointestinal duplication (GD) is a rare congenital disorder [1]. Gastric duplication cysts (GDCs) are a rare diagnosis in adults and are more frequently seen in young children [2]. Several theories have been proposed for the formation of GDCs [1]. It is generally asymptomatic in adults and discovered fortuitously during endoscopy, radiological examination, or laparotomy [3]. Diagnostic confirmation is based on imaging examinations, including ultrasound, computed tomography (CT), and magnetic resonance imaging (MRI) [1], and endoscopic ultrasound (EUS). However, the preoperative diagnosis of GDCs is difficult [4]. This case describes a fortuitous discovery of a noncommunicating GDC in an adult after an endoscopic ultrasound-guided fine-needle aspiration (EUS-FNA) of a suspected mucinous cystadenoma of the pancreas. This case report aims to increase the knowledge to distinguish GDC from a pancreatic mucinous cystadenoma and to understand that the split aspect of the gastric wall in GD can be noted on EUS after guided fine-needle aspiration (FNA).

## Case presentation

**Figure 1:**
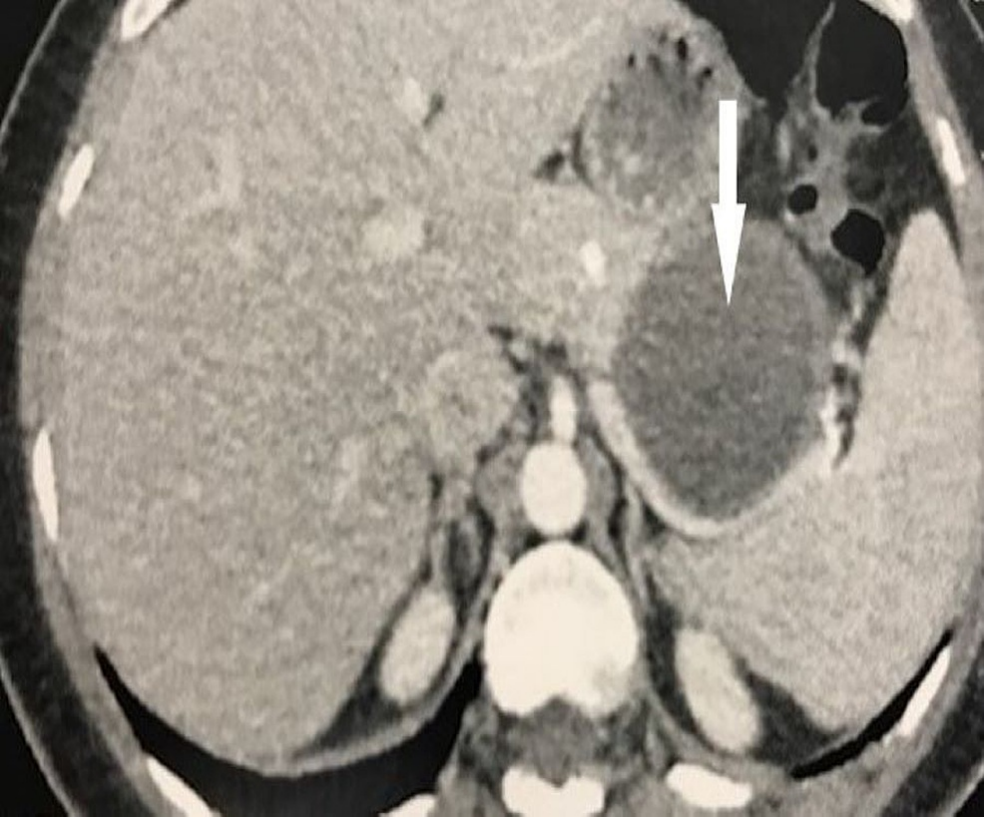
A uro-CT suggesting a cystadenoma of the tail of the pancreas. CT: computed tomography

A 34-year-old female with no significant medical history presented with renal colic and no other digestive or extradigestive signs. Her abdominal examination was normal. The patient underwent abdominal ultrasonography that showed a cystic image at the left lumbar. Subsequently, she underwent an uro-CT which revealed a cystic, thick-walled, slightly enhanced lesion of the tail of the pancreas, measuring 64 × 59 × 53 mm, with a homogeneous fluid content suspected to be a cystadenoma (Figure 1). The patient was then referred to our service.

**Figure 2:**
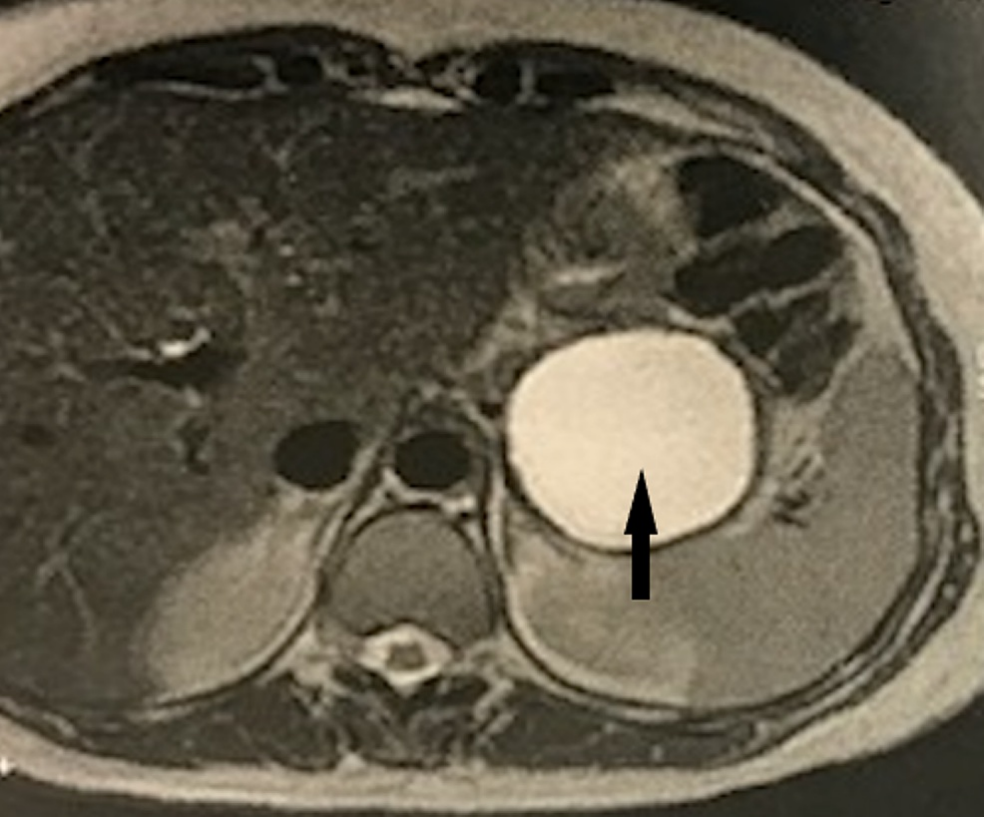
A pancreatic MRI suggesting a mucinous cystadenoma of the tail of the pancreas. MRI: magnetic resonance imaging

**Figure 3:**
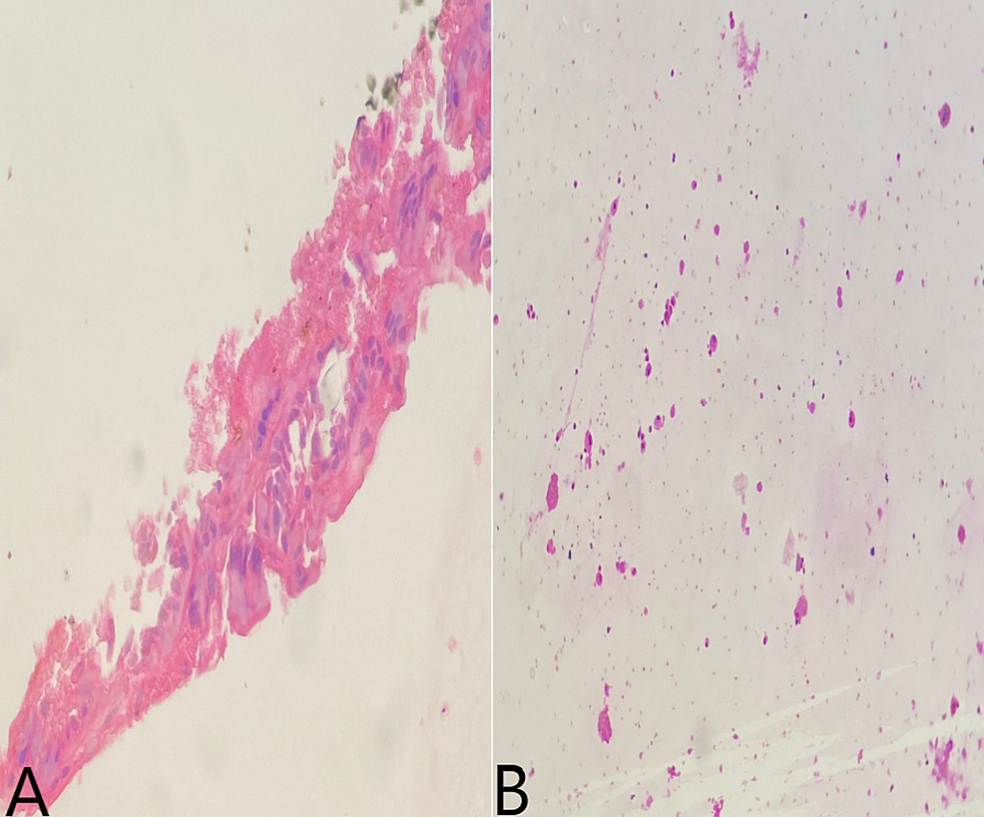
The pathology showing a cystic or pseudocyst-like lesion. A: A flap of a regular columnar epithelium (hematoxylin and eosin stain); B: inflammatory cytology rich in histiocytes (pink stain).

The biological examination was normal. The pancreatic MRI showed a 57 × 60 mm unilocular and homogenous thick-walled cystic lesion of the pancreatic tail, suggesting a mucinous cystadenoma (Figure 2). EUS showed a cystic thick-walled formation in the tail of the pancreas, measuring 50 mm on the major axis. After EUS-FNA, a split aspect of the gastric wall appeared, evoking a GDC. The liquid was nonviscous. The pathology showed a cystic or pseudocyst-like lesion with a nondense mucinous base, not characteristic of a mucinous cyst, with the presence of siderophages and rare glandular cells (Figure 3). Three years later, the patient remains asymptomatic with no gastrointestinal symptoms.

## Discussion

Duplication of the gastrointestinal tract (GIT) may occur from the oral cavity to the anus [4]. It is most commonly located in the ileum, followed by the esophagus, jejunum, colon, stomach, and appendix [5]. Although it can be found anywhere in the stomach [2], it is mainly located in the greater curvature [3]. GD accounts for 2‑8% of all GIT duplication deformities. Moreover, it most frequently affects females [1].

GD can be cystic or tubular and may or may not communicate with the GIT. The duplication cyst is the noncommunicating cystic form, which is the most common [6]. In our case, we noted a noncommunicating GDC. Duplications result from disturbances in embryonic development [5]. Various congenital anomalies may be observed such as esophageal diverticulum, intestinal atresia, or spinal deformity [4].

GDC may be asymptomatic or symptomatic [1]. A very small subset of patients can remain asymptomatic and are diagnosed incidentally, as observed in our patient. GDCs may also present with various digestive symptoms, including epigastric fullness, vague abdominal pain, dyspepsia, abdominal distention, nausea, vomiting, weight loss, anemia, and dysphagia [2]. Its complications include intestinal bleeding such as hematemesis and melena, which rarely occur [7,8]. Other complications are adhesive obstruction and perforation, which are caused by the mucosa corrosion effect from ectopic gastric acid secretion [4].

Because the preoperative diagnosis of GDCs is difficult, they are usually discovered intraoperatively in adults [5]. Diagnostic investigations include ultrasound, CT, MRI [1], and EUS. Typically, GDCs present on CT as a thick-walled cystic lesion with an enhancement of the inner lining. Calcification can also be observed. Mucinous cystic tumors of the pancreas have similar CT features as GDCs adjoining the pancreas, which makes them indistinguishable on CT. Moreover, when the wall is thin, there is no enhancement of the inner cyst [5]. Compared to CT, MRI can provide more information about the content of the cyst. In GDCs, the type of fluid is different in each case according to infection, bleeding, or chronic inflammation. Therefore, MRI is not efficient in diagnosing GDC [5]. EUS is the best modality to help clarify diagnosis and can differentiate between intramural and extramural gastric lesions. In a cyst with a hypoechoic intermediate muscular layer and an echogenic internal mucosal layer, the diagnosis of GDC is highly possible [5]. Because cytological features of GDC and mucinous pancreatic neoplasms may be similar, the role of EUS-FNA in diagnosing GDC is uncertain [3].

For diagnosing a GDC, the requisite criteria are: (1) the wall of the cyst and the stomach are contiguous; (2) a smooth muscle circumscribes the cyst and is continuous with the muscle of the stomach; and (3) a GIT epithelium lines the wall of the cyst [5]. The differential diagnoses of GD included adrenal cyst, stomach adenoma, and pancreatic cyst [1].

Malignant transformation of GDC is very rare [6]. It mostly transforms into adenocarcinoma, and rarely into neuroendocrine tumors, squamous cell carcinoma, and GIT tumors. Initial diagnosis of malignant transformation can be made by CT, and endoscopy can distinguish the gastric mass [9]. The mechanism of malignant transformation remains unclear, and further investigations may improve our understanding of malignant potential [4].

There are several treatment modalities, including endoscopic removal, enucleation, and cyst gastrostomy. The surgical excision of the cyst is considered the mainstay of therapy [10]. Partial gastrectomy with or without distal pancreatectomy, depending on the site of the duplication, are other treatment options [1,6,10]. Because surgical treatment avoids the risk of malignancy and possible complications [5], it should be performed when the patient is symptomatic or when the risk of malignant degeneration is high [2].

## Conclusions

GDCs are extremely rare and their presentation in adults is even rarer. Frequently, they remain asymptomatic but can present with various digestive symptoms. GDCs are usually diagnosed intraoperatively in adults. Preoperative diagnosis is usually done by imaging. With this case report, we can confirm that GDCs can remain asymptomatic in adults and that CT and MRI findings may be more consistent with a pancreatic cyst. In our case, the diagnosis of GDCs was confirmed only after EUS-FNA by showing a split aspect of the gastric wall.
